# Case report: Denosumab-associated acute heart failure in patients with cardiorenal insufficiency

**DOI:** 10.3389/fendo.2022.970571

**Published:** 2022-09-14

**Authors:** Yuexian Xing, Sicong Ju, Mengyu Sun, Shoukui Xiang

**Affiliations:** Department of Endocrinology, the Third Affiliated Hospital of Soochow University, Changzhou, China

**Keywords:** denosumab, heart failure, hypocalcemia, cardiorenal insufficiency, osteoporosis

## Abstract

Denosumab is a pivotal treatment for postmenopausal women with osteoporosis. Although its clinical use is generally well tolerated by patients, denosumab in patients with renal insufficiency may increase the risk of hypocalcemia. Thus, we have to consider the population of denosumab in the treatment of osteoporosis and preventive measures for related complications. In a patient with cardiorenal insufficiency, we reported a case of denosumab-induced hypocalcemia complicated by acute left heart failure due to delayed administration of active vitamin D and calcium supplements. The patient’s symptoms did not improve after anti-heart failure treatment. However, after adequate calcium and vitamin D supplementation subsequently, the patient’s symptoms of heart failure were rapidly relieved, and the serum calcium level returned to normal within three weeks. Therefore, our case showed that the application of denosumab in patients requires assessment of cardiac and renal function, timely calcium and vitamin D supplementation, and enhanced monitoring of serum calcium levels to prevent acute left heart failure induced by denosumab-related hypocalcemia.

## Introduction

Denosumab is a human recombinant monoclonal antibody that prevents the binding of nuclear factor kappa-B ligand (RANKL) to receptors on osteoclasts (1), thereby inhibiting osteoclast activity. Denosumab has been approved for osteoporosis treatment, including in patients with renal insufficiency (2). Hypocalcemia, a side effect of denosumab, occurs in about 14% of patients and is more frequent in patients with chronic kidney disease (CKD) (3, 4). Although gradually gaining attention, it remains to be observed whether denosumab-induced hypocalcemia will lead to more serious complications.

Calcium plays an important role in cardiac muscle contraction and cell metabolism. Although hypocalcemia has been shown to lead to cardiac decompensation in animals, heart failure due to hypocalcemia is quite rare in clinical practice. According to previously, hypocalcemia accompanied by renal insufficiency, can induce pulmonary edema and cardiac insufficiency (5). Moreover, hypocalcemia is an independent predictive factor for left ventricular diastolic dysfunction in patients with CKD (6). The case described here is that of hypocalcemia complicated by acute left heart failure occurring after a single dose of denosumab in a patient with cardiorenal insufficiency.

## Case description

An 86-year-old female with heart failure and grade four cardiac function presented to our hospital with dyspnea, coughing and phlegm that she had been experiencing for two days. The patient reported paroxysmal nocturnal dyspnea, nausea, and oliguria but otherwise had no complaints. The patient suffered from chronic cardiac insufficiency for 1 year and grade two cardiac function. Moreover, the patient had a history of hypertension for 10 years, oral nifedipine antihypertensive therapy. In addition, she had a history of coronary atherosclerosis for 10 years, chronic renal insufficiency due to chronic nephritic syndrome for 2 years, anemia for 2 years, and carotid plaque (unknown course). Physical examination revealed a coarse breath sounds, a few moist rales at the base of both lungs, arrhythmia. There were hands twitching during blood pressure measurement and no other abnormalities.

Laboratory findings revealed a serum NT-proBNP 20800 ng/L (300-450 ng/L), blood urea nitrogen of 4.93 mmol/L (2.5-6.1 mmol/L), creatinine of 102.9 μmol/L (46-92 μmol/L, estimated glomerular filtration rate of 42.26 mL/min/1.73 m^2^), serum calcium of 1.01 mmol/L (corrected to 1.20 mmol/L for hypoalbuminemia), parathyroid hormone (PTH) of 298 pg/mL (15-65pg/mL), serum 25-OH vitamin D of 8.4 ng/mL, arterial pH of 7.41 (7.35-7.45), ionized calcium of 0.52 mmol/L (1.12-1.32 mmol/L). The echocardiography showed that left ventricular dysfunction, the value of EF is 51% (53%-75%), left ventricular muscle thickening. The electrocardiographic showed that sinus rhythm, premature atrial contractions, poor R-wave progression, and QTc prolongation. The patient was treated with furosemide, spironolactone, antihypertensive, antiplatelet and intravenous calcium gluconate infusion. However, the patient’s heart failure symptoms and various indicators did not improve significantly.

**Figure 1:**
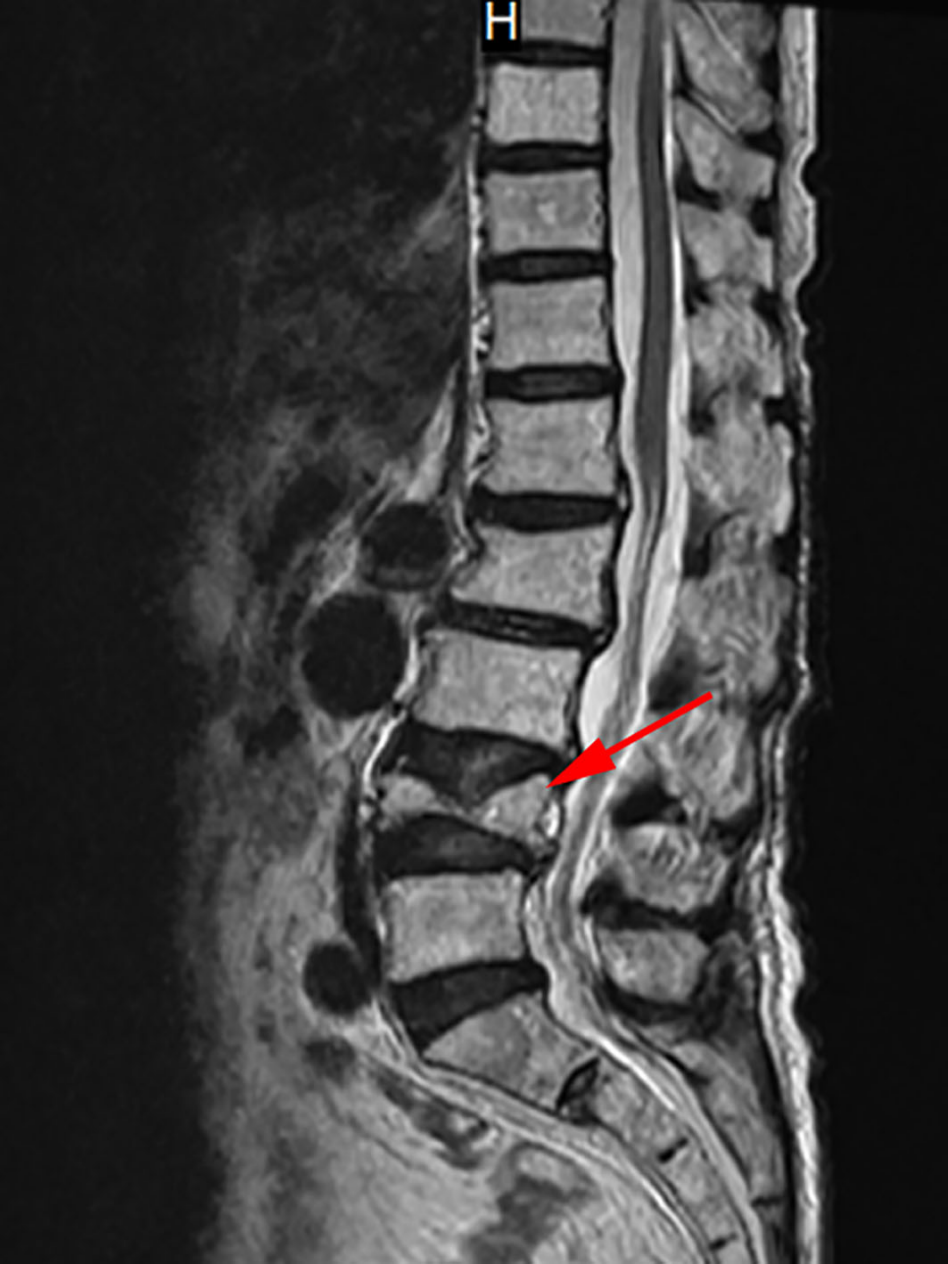
MRI of the lumbar vertebra. This image shows the MRI of the patient’s lumbar vertebra. The red arrow marks the 4th lumbar vertebra with a compression fracture.

Therefore, the Department of Cardiovascular Medicine asked the Department of Endocrinology for a suggestion, considering recalcitrant hypocalcemia may have affected the management of heart failure. Reviewing her medical history, the patient presented to the spine surgery two weeks ago with lumbago and pain in both lower extremities that she had been experiencing for 1 year. Physical examination revealed a percussion tenderness on the protuberance of the 2nd and 3rd lumbar vertebrae, no radiating pain to the lower limbs, straight leg raising test (-), leg strengthening test (-), no abnormal muscle tension, and good peripheral blood supply of the limbs. In the absence of a history of trauma, magnetic resonance imaging (MRI) of the lumbar vertebra showed a compression fracture of the 4th lumbar vertebra and mild deformation of the 2nd lumbar vertebra (**Figure 1**). The spine surgeon checked the serum calcium and phosphorus, both within the normal range. Then the surgeon recommended bed rest and anti-osteoporotic therapy with denosumab 60mg ih, without calcium and vitamin D supplementation.

**Figure 2:**
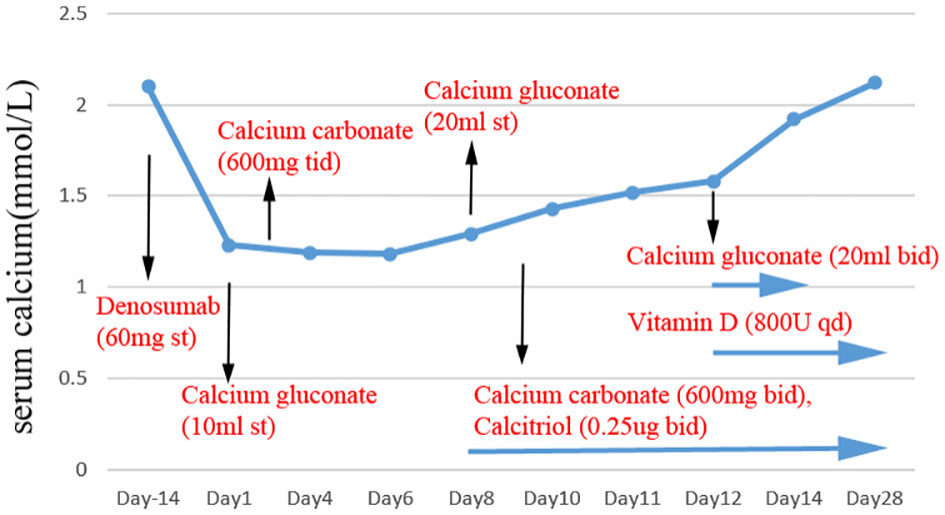
The level of serum calcium after denosumab administration. Black arrows mark the time of denosumab, calcium gluconate, calcium carbonate, vitamin D and calcitriol supplementation.

Due to the patient’s abnormal renal function, we considered that denosumab might have induced hypocalcemia and, therefore, acute heart failure. Thus, we recommended that the patient supplement calcium carbonate 600mg bid, vitamin D 800U qd, and calcitriol 0.25ug bid combined with anti-acute heart failure therapy. Four days later, the patient’s serum calcium increased to 1.72 mmol/L (corrected to 1.90 mmol/L for hypoalbuminemia), urinary calcium of 2.95 mmol/d (2.5-7.5mmol/d), the symptoms of heart failure were relieved and the level of heart failure markers decreased, significantly. Then she left the hospital. After two weeks, the patient’s family said that the serum calcium and PTH had returned to normal levels (**Figure 2**), and the patient had no complaints of discomfort. Subsequently, we instructed the patient to start to reduce calcium carbonate to 600 mg qd, and visit the outpatient clinic two weeks later to adjust the calcium supplementation according to the serum calcium level.

## Discussion

This case report indicated that the risk of denosumab-induced acute heart failure related to hypocalcemia may be increased in patients with cardiorenal insufficiency. There have been no reports of acute heart failure following the treatment of osteoporosis with denosumab.

Denosumab is a human IgG2 monoclonal antibody that inhibits osteoclast activation by interacting with receptor activator of nuclear factor-kappaB (RANK) ligand (RANKL) to prevent its binding to the RANK receptor in osteoclasts (7). In clinical work, denosumab is often used to treat osteoporosis in postmenopausal women (8), and the usual dose is 60 mg subcutaneously, administered once every 6 months. Hypocalcemia is a common side effect of denosumab due to serum calcium may be reduced by decreasing bone resorption and increasing bone mass during denosumab. If the patient has hypocalcemia before the treatment, the hypocalcemia needs to be corrected, and the serum calcium level must be closely observed during the treatment. Calcium and vitamin D should be supplemented, especially within a few weeks of starting treatment.

Although the risk of hypocalcemia associated with denosumab has been described in the previous (9, 10), it especially appears to be higher in patients with CKD (11–13). In patients with renal failure, due to reduced intestinal calcium absorption, patients gradually exhibit a state of high bone turnover, which maintains serum calcium by transferring skeletal calcium into the circulation. In patients with CKD, treatment with denosumab for osteoporosis or high bone turnover results in a decrease in bone resorption followed by a sustained decrease in serum calcium in the absence of adequate calcium supplementation. In our case, the patient had chronic renal insufficiency. With a history of CKD, a possible abnormal bone resorption could not be excluded, masking the presence of hypocalcemia. The lack of adequate early calcium supplementation in this patient resulted in severe hypocalcemia during the treatment with denosumab for osteoporosis. Importantly, the patient experienced a significant decrease in serum calcium levels before and after treatment with denosumab, so we considered that the main reason for the hypocalcemia was the application of denosumab. The incidence of hypocalcemia in hospitalized patients is about 27.72%, mainly in people older than 65 years old (14). This data reminds us that we need to be especially vigilant about the occurrence of hypocalcemia and the supplementation of calcium before the application of denosumab, especially in elderly patients.

The RANKL/RANK/OPG pathway is involved in the pathogenesis of both osteoporosis and cardiovascular disease (15–17), suggesting that there may be a close relationship between osteoporosis and cardiovascular disease. Thus, it is significant to explore the effect of denosumab on cardiovascular outcomes in patients with osteoporosis. However, the relationship between the two is currently unclear. Although systematic reviews have identified cardiovascular adverse events in post-menopausal women with osteoporosis or low BMD treated with denosumab were more frequently compared with bisphosphonates, there is no significant difference in placebo group (17, 18).

In this case, we found that denosumab may induce acute heart failure through hypocalcemia. As described above, the patient has chronic renal insufficiency. Although denosumab is not metabolized through the kidneys, the incidence of hypocalcemia is significantly higher in CKD (3, 4). According to Catalano A, et al., hypocalcemia accompanied by renal insufficiency can induce cardiac insufficiency or heart failure (5, 19, 20). Heart failure due to hypocalcemia is often associated with idiopathic or postoperative hypoparathyroidism and vitamin D deficiency (21). In our case, hypocalcemia was accompanied by elevated PTH, suggesting that hyperparathyroidism was secondary and that the development of acute heart failure was not associated with hypoparathyroidism. Thus, hypocalcemia was mainly caused by the side effects of denosumab in the treatment of osteoporosis. In cardiomyocytes, calcium functions as a direct central mediator of electrical activation and ion channel gating, playing a key role in the mediation of excitation-contraction coupling. Due to the history of cardiac insufficiency, the symptoms of acute heart failure in the patient were relieved by the correction of serum calcium levels, without cardiac function fully recovered.

## Conclusion

Reducing the risk of denosumab-induced hypocalcemia and consequent more serious complications is significant. Our observations emphasize the need for careful assessment of cardiac function in CKD patients before denosumab exposure, reinforcement calcium supplementation and close monitoring of serum calcium levels subsequently.

## Data availability statement

The original contributions presented in the study are included in the article/**Supplementary Material**. Further inquiries can be directed to the corresponding author.

## Ethics statement

Written informed consent was obtained from the individual(s) for the publication of any potentially identifiable images or data included in this article.

## Author contributions

XY edited the manuscript. JS and SM collected clinical data. XS guided the writing ideas. All authors contributed to the article and approved the submitted version.

## Funding

The authors are grateful to support from the Changzhou Key Research and Development Project (CE20205022), a Major Project of the Changzhou Health Commission (ZD201903).

## Conflict of interest

The authors declare that the research was conducted in the absence of any commercial or financial relationships that could be construed as a potential conflict of interest.

## Publisher’s note

All claims expressed in this article are solely those of the authors and do not necessarily represent those of their affiliated organizations, or those of the publisher, the editors and the reviewers. Any product that may be evaluated in this article, or claim that may be made by its manufacturer, is not guaranteed or endorsed by the publisher.
